# INSM1 Expression in Mesenchymal Tumors and Its Clinicopathological Significance

**DOI:** 10.1155/2022/1580410

**Published:** 2022-12-07

**Authors:** Qian Zhang, Yuting Dong, Meidong Zhou, Yujuan Guo, Liping Lou, Zhiling Qu, Yiyun Zheng, Yaqi Duan

**Affiliations:** ^1^Institute of Pathology, Tongji Hospital, Huazhong University of Science and Technology, Wuhan 430000, China; ^2^Department of Pathology, School of Basic Medical Science, Huazhong University of Science and Technology, Wuhan 430000, China

## Abstract

**Background:**

Insulinoma-associated protein 1 (INSM1) has been identified as a nuclear marker of neuroendocrine tumors. Although INSM1 appears to be a subtle and specific biomarker for neuroendocrine tumor, its expression and clinicopathological significance in mesenchymal tumors remain unclear.

**Methods:**

We analyzed INSM1 mRNA level in GEO database and conducted immunohistological staining to detect the expression of INSM1 on 576 mesenchymal tumors from pathology department of Tongji Hospital.

**Results:**

At transcription level, INSM1 expression in AITL (angioimmunoblastic T-cell lymphoma) was higher than their adjacent normal tissues as well as Hodgkin's lymphoma. Moreover, INSM1 expression in well-differentiated liposarcoma (WDLPS) was significantly higher than normal fat (*P* = 0.014) and dedifferentiated liposarcoma (DDLPS) (*P* = 0.0248). At protein level, the positive rate of INSM1 in AITL was 18/48 (47.4%), while in DDLPS was 9/20 (45%). INSM1 expression in AITL was significantly higher than Hodgkin's lymphoma (*P* = 0.008). And INSM1 expression in WDLPS was significantly lower than DDLPS (*P* = 0.015).

**Conclusion:**

The combination of GEO data and immunohistochemistry data indicated that the expression level of INSM1 was higher in AITL compared with normal control, suggesting that INSM1 may be involved in pathogenesis of AITL. The abnormal expression of INSM1 was found in WDLPS, and the positive rate of INSM1 was higher in DDLPS than in WDLPS. INSM1 may be involved in the regulation of liposarcoma development. There were significant differences in the expression of INSM1 between AITL and Hodgkin's lymphoma and WDLPS and DDLPS. These findings may assist in the differential diagnosis of these tumors when common markers are difficult to identify, enriching the diagnostic index system of mesenchymal tumors.

## 1. Introduction

Insulinoma-associated 1 (INSM1) gene is intronless and encodes a protein containing both a zinc finger DNA-binding domain and a putative prohormone domain. This gene is a sensitive marker for neuroendocrine differentiation of human lung tumors. INSM1 is involved in the regulation of a variety of downstream signaling pathways, including Sonic Hedgehog, PI3K/AKT, MEK/ERK1/2, ADK, p53, Wnt, histone acetylation, LSD1, cyclin D1, Ascl1, and N-MyC pathways [1]. SNAG motifs of INSM1 are involved in cell cycle arrest and recruit histone modifiers in posttranscriptional modification: histone deacetylase 12 (HDAC1/2) and REST corepressor 1-3 (RCOR1-3). Proline-rich region of INSM1 can bind to cyclin D1, resulting in cell cycle arrest. Moreover, INSM1's binding to RACK1 enhances the effect of insulin receptor-mediated signaling pathway. Prohormone dibasic conversion sites are characteristic sites required for prohormone convertase processing of polypeptide hormones such as insulin, glucagon, growth hormone, and pancreatic polypeptide [2–8].

INSM1 reduced significantly in the late development of the human brain and pancreas and completely disappears in normal adult tissues. However, it is reexpressed in neuroendocrine tumors. It can be considered that the “reexpression” of INSM1 mimics the dedifferentiation event of normal embryonic development [9–11]. Reactivation of INSM1 has been observed in insulinoma, pheochromocytoma, pituitary tumor, medullary carcinoma of the thyroid, medulloblastoma, neuroblastoma, retinoblastoma, small-cell lung cancer, and neuroendocrine tumors of the head and neck/skin/prostate/female genital tract [3, 12–19].

Some sporadic case studies have shown that neuroendocrine indicators can also be expressed in soft tissue tumors, such as synapsin (Syn) and chromogranin A (CgA) expressed in extrasolar myxochondrosarcoma, alveolar rhabdomyosarcoma, and angiosarcoma [20–24]. As an emerging neuroendocrine marker, whether INSM1 is beneficial to diagnose soft tissue tumors with neuroendocrine differentiation aroused the interest of some researchers. It has been found that INSM1 is positively expressed in 90% of extracorporeal myxochondrosarcoma [25]. INSM1 is expected to become a potential marker for the diagnosis of this tumor. Although the proportion was only 26% in angiosarcomas, we cannot ignore the presence of angiosarcomas with abnormal INSM1 expression, especially when evaluating poorly differentiated and inadequately sampled samples [25, 26]. In addition, a case study reported nuclear translocation t(12; 14) (q23.2; Q32.3) may make ASCL bind to INSM1 enhancer, upregulating INSM1 expression in chronic lymphocytic leukemia. This also leads us to explore the possibility of “reexpression” of INSM1 in blood diseases [27].

Although INSM1 appears to be a subtle and specific biomarker for neuroendocrine tumors, its expression in mesenchymal tumors and its clinicopathological significance are still unclear [28]. Therefore, whether INSM1 can continue to maintain excellent diagnostic performance in mesenchymal tumors is indeed a question worth exploring. Although some researchers have added mesenchymal tumors in the study of neuroendocrine tumors, more tissue types have not been involved. The study on the expression of INSM1 in mesenchymal tumors is still limited. Here, we use immunohistochemical method to detect the expression of INSM1 in 576 mesenchymal tumors. In addition, we also used R language to analyze GEO databases due to limited samples in our hospital to evaluate its potential value in clinical diagnosis and treatment.

## 2. Materials and Methods

### 2.1. Tumor Specimens

Three pathologists rereviewed the tumors and selected 576 formalin-fixed paraffin embedded (FFPE) samples of mesenchymal tumors from the Institute of Pathology, Tongji Hospital, Tongji Medical College, Huazhong University of Science and Technology. Cases of puncture specimen, being too small to be sectioned again, or having two or more pathologically diagnosed tumors, were excluded in this study.

### 2.2. Immunohistochemistry

Tissue sections were deparaffinized and hydrated, and endogenous peroxidase activity was blocked. Antigen retrieval was achieved using high pH antigen repair fluid DM828 (Dako Denmark A/S) in a PT-Link set at 98°C for 25 min. The whole tissue sections were incubated with anti-INSM1 (1 : 200; A-8, Santa Cruz Biotechnology, USA) for 30 min at room temperature. Immunostaining was achieved by an enzyme-conjugated polymer complex (Dako K8002) adapted for an autostainer (Dako Autostainer Link 48). Nuclear staining of the small intestine was used as a negative control and known positive small-cell lung cancer as positive control. The number of positive cells in the tumor area/the total number of cells in the tumor area was less than 5%, which was considered negative.

### 2.3. GEO Database Analysis

R package limma/ggplot package and the *T* test method were used to confirm the INSM1 expression in mesenchymal tumors at transcriptional level. The transcriptomic data were downloaded from GEO (https://www.ncbi.nlm.nih.gov/geo/) including dataset which is shown in Supplementary materials Table S1.

### 2.4. Data Statistics and Analysis

The results of INSM1 in public database were analyzed by *T* test using R language; immunohistochemical results were analyzed by the SPSS21.0 software and GraphPad Prism7.0. The chi-square or Fisher test was used to compare the positive rate difference of INSM1 in different tumors, and *P* < 0.05 was considered statistically significant.

## 3. Results and Discussion

### 3.1. INSM1 mRNA Level of Mesenchymal Tumors in GEO Database

**Figure 1:**
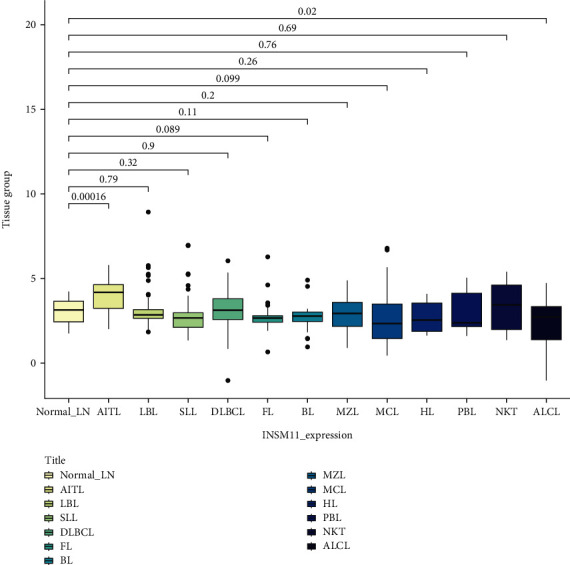
The mRNA level of INSM1 in lymphoma was analyzed by GEO (normal lymph node as control).

**Figure 2:**
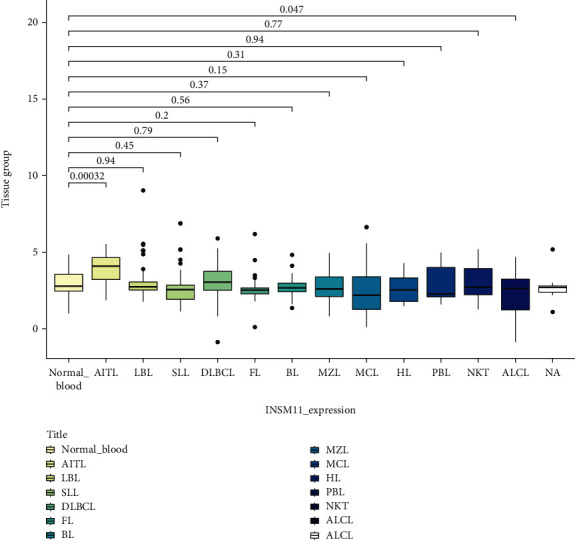
The mRNA level of INSM1 in lymphoma was analyzed by GEO (normal peripheral blood as control).

**Figure 3:**
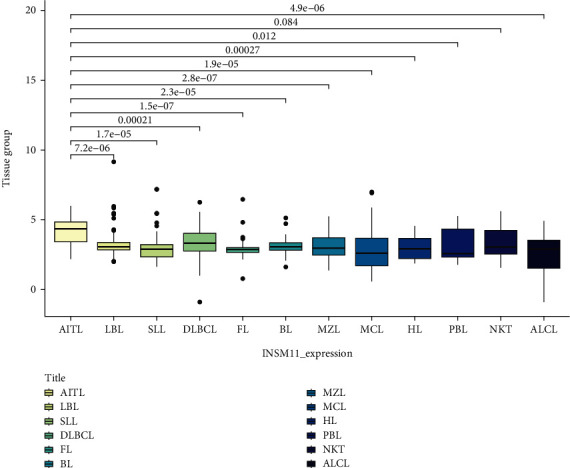
The mRNA level of INSM1 in lymphoma was analyzed by GEO (AITL as control).

Data analysis showed that the expression of AITL and ALCL was different from that of normal lymph node/peripheral blood. The INSM1 mRNA of AITL was higher than that of normal control, and the INSM1 mRNA of ALCL was lower than that of normal control. The INSM1 mRNA of AITL was significantly higher than that of most lymphomas (there was no statistically significant difference between AITL and NK/T-cell lymphoma) (Figures 1–3). The abbreviations of hematologic neoplasms are as shown in Table 1. Other lymphoma pairwise comparison is shown in Table S2.

**Figure 4:**
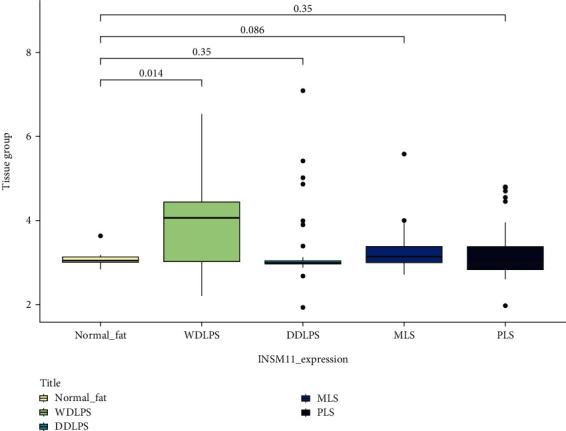
The expression of INSM1 in liposarcoma was analyzed by GEO. WDLPS: well-differentiated liposarcoma; DDLPS: dedifferentiated liposarcoma; MLS: myxoid liposarcoma; PLS: pleomorphic liposarcoma.

INSM1 mRNA level between multiple subtypes of soft tissue tumor showed significant differences (Table S3). Many tumor types were lack of corresponding normal control. Thus, we focus on liposarcoma, rhabdomyosarcoma, and bone tumor. We found that the INSM1 mRNA content in WDLPS was significantly higher than that in normal fat, while there was no significant difference in the INSM1 mRNA level between bone tumor/rhabdomyosarcoma compared with their normal control (Figure 4 and Figure S1, 2).

### 3.2. INSM1 Expression in Lymphoma at Protein Level

**Figure 5:**
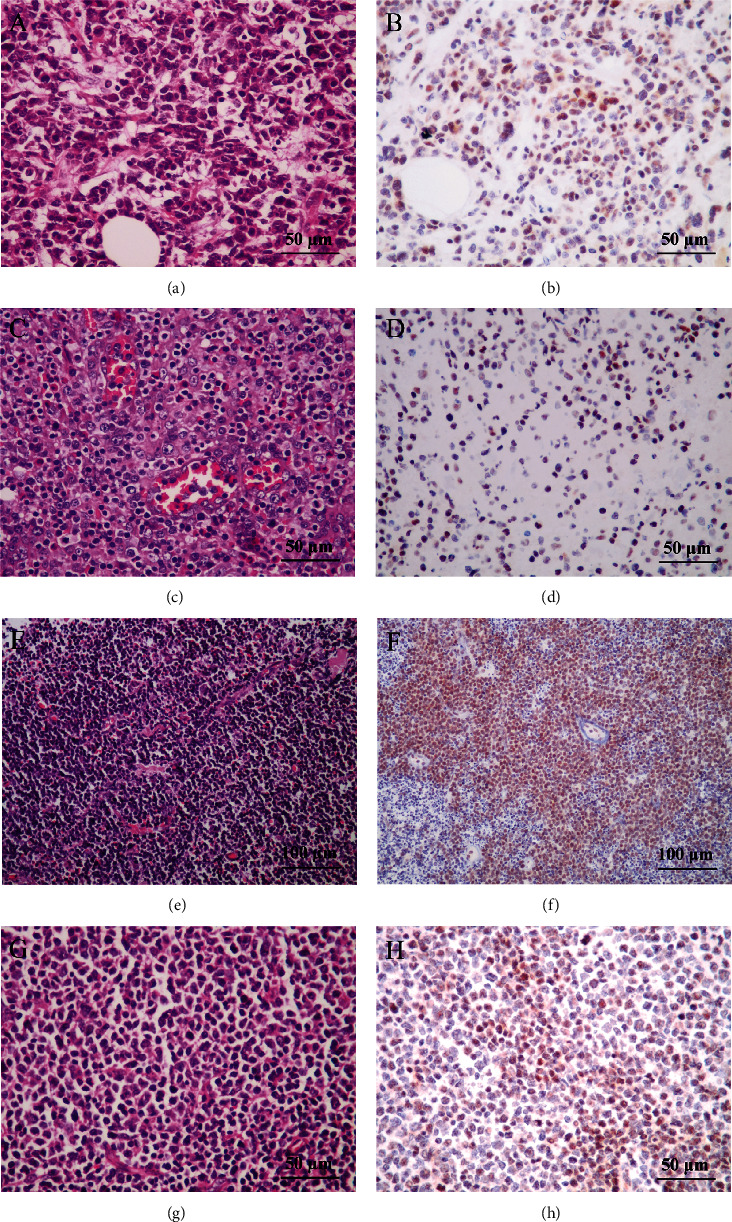
Representative HE and IHC staining of INSM1 in PBL, AITL, BL, and FL. (a, b) Plasmablastic lymphoma (PBL): the nucleus is large and deviated from the cell center, the cytoplasm is eosinophilic or basophilic, perinuclear clarity, and mitotic pattern is common, INSM1+ (400x); (c, d) vascular immunoblastic T-cell lymphoma (AITL): microscopically, there are dense medium-sized nuclei with abundant and bright cytoplasm around the nuclei and small blood vessels between the tumors, INSM1+ (400x); (e, f) Burkitt's lymphoma: diffuse growth of homogeneous neoplastic cells with a small number of histiocytes with phagocytic debris, INSM1 (200x); (g, h) follicular lymphoma (FL): here are small to moderate tumor cells with irregular nuclei and no distinct nucleoli, INSM1+ (400x).

**Figure 6:**
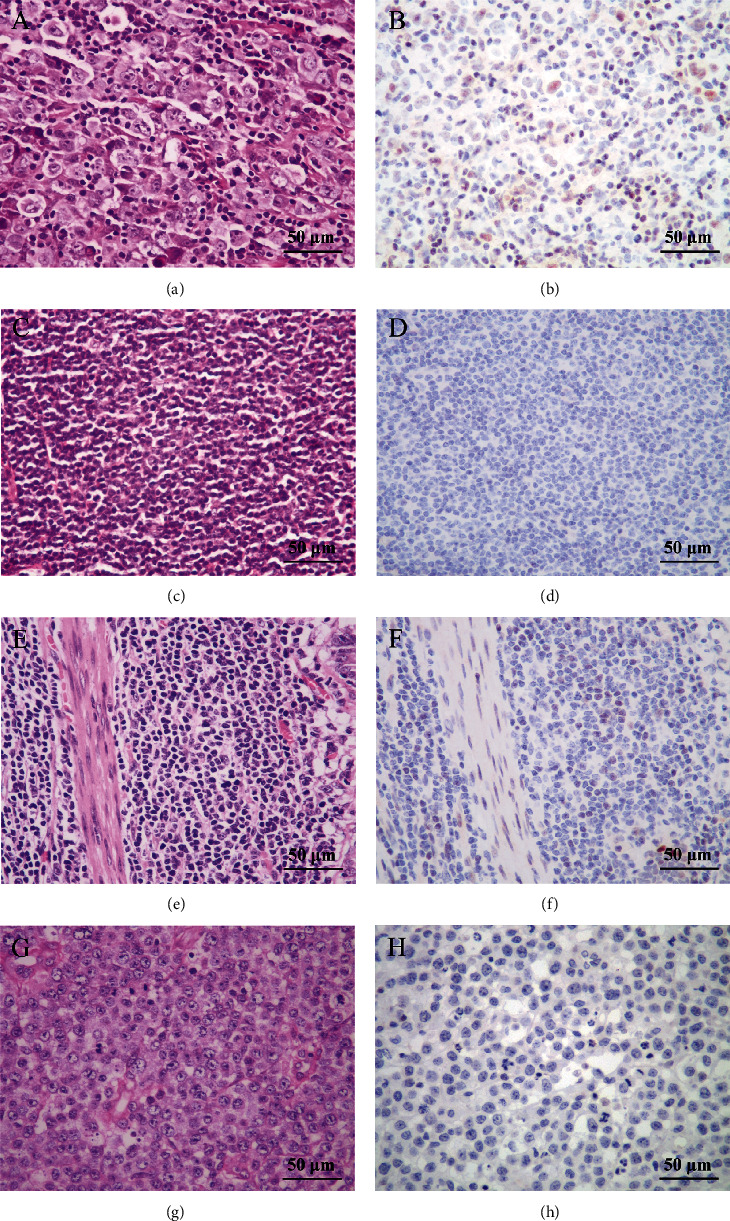
Representative HE and IHC staining of INSM1 in HL, SLL, ALCL, and NKT lymphoma. (a, b) Hodgkin's lymphoma: R-S cells were scattered in inflammatory background cells, INSM1+ (400x); (c, d) small lymphocytic lymphoma (SLL): round dark nucleus without obvious nucleoli, INSM1- (400x); (e, f) NK/T-cell lymphoma: the tumor is dominated by small cells, and partial nuclear staining is deep, INSM1- (400x); (g, h) anaplastic cell lymphoma (ALCL): intercellular.

We found that a positive rate of INSM1 in plasmablastic lymphoma was 50%. However, due to the small number of samples, whether this type of tumor really has such a high positive rate awaits further expanding sample analysis. The positive rate of INSM1 in AITL was 18/38 (47.4%). The positive rate of follicular lymphoma and Burkitt's lymphoma was more than 30%. INSM1 positive rates range from 10% to 30% including marginal zone lymphoma, diffuse large B-cell lymphoma, mantle cell lymphoma, and T/B lymphoblastic lymphoma. INSM1 protein was not expressed in small lymphocyte lymphoma, NK/T-cell lymphoma, and anaplastic large cell lymphoma (Table 2). The HE and IHC staining of each lymphoma is shown in Figures 5 and 6 and Figure S3.

**Figure 7:**
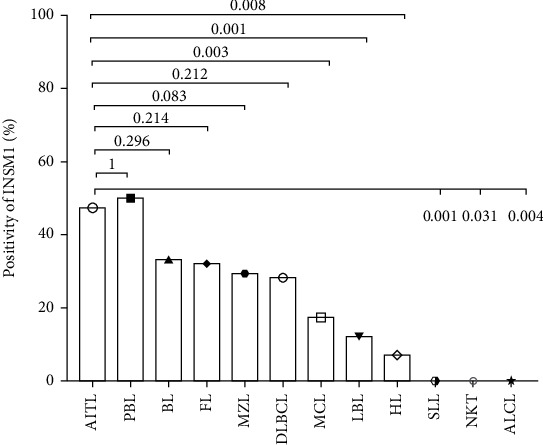
Differences in INSM1 protein expression between AITL and other lymphomas. The abbreviations of hematologic neoplasms are as shown in Table 1.

Immunohistochemical staining showed that the level of INSM1 protein in AITL was significantly higher than that in many other lymphomas, consistent with in GEO database analysis. As mentioned above, we found significant differences in INSM1 mRNA expression levels between AITL and other lymphomas except for NK/T-cell lymphoma. We validated AITL at the protein level and found significant differences between AITL and Hodgkin's lymphoma, T/B lymphoblastic lymphoma, mantle cell lymphoma, small lymphocyte lymphoma, NK/T-cell lymphoma, and anaplastic large cell lymphoma (Figure 7). It is partly consistent with the results of bioinformatics analysis.

### 3.3. INSM1 Expression in Liposarcoma at Protein Level

**Figure 8:**
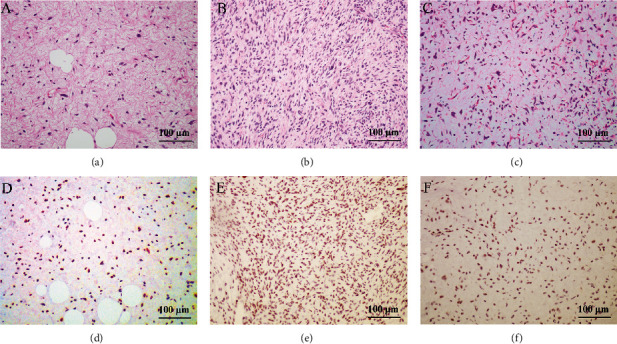
Positive expression of INSM1 in liposarcoma. (a, d) Highly differentiated liposarcoma: there are scattered hyperchromatic heteromorphic cells in the fibrous collagen stroma, marked fibrous septa, hyperchromatic nuclei, and enlarged spindle cells. INSM1 staining was positive in the tumor region. Tumor cells were scattered in the mucus matrix. (b, e) Dedifferentiated liposarcoma: the cells are disordered, some of them are fasciculate/lumpy, and the nuclei of tumor cells are hyperchromatic. (c, f) Myxoid liposarcoma: tumor cells are seen scattered along a curvilinear network of thin-walled blood vessels in the mucinous matrix (200x).

**Figure 9:**
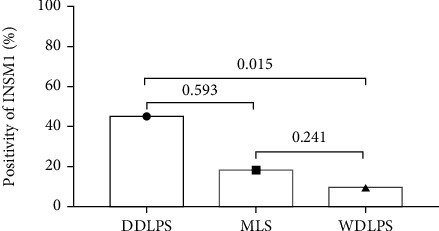
Differences in INSM1 protein expression among different subtypes of liposarcoma.

The positive rates of INSM1 in soft tissue tumors are listed in Table 3. The representative HE and IHC staining of each soft tissue tumor is shown in Figure 8 and Figure S4–7. According to GEO analysis mentioned above, we further explored INSM1 expression rates between dedifferentiated liposarcomas (DDLPS) and other subtypes of liposarcomas. Positive rates of INSM1 in DDLPS were significantly higher than well-differentiated liposarcoma (WDLPS) (*P* = 0.015) (Figure 9), while GEO analysis showed that DDLPS was significantly lower than WDLPS (*P* = 0.0239) in terms of INSM1 transcription level. INSM1 mRNA was not significant between DDLPS and myxoid liposarcoma (MLS), which was consistent with our immunochemical results (Figure S1, 2).

## 4. Discussion

Insulinoma-associated protein 1 (INSM1) has been considered as a novel immunostaining marker for neuroendocrine tumors (NETs) and is hypothesized to be more reliable than first-generation NET biomarkers, such as CGA (chromogranin A), SYP (synaptophysin), and CD56 (neural cell adhesion molecule). Meanwhile, INSM1 expression in non-NETs was relatively less studied. As previously reported, solitary fibrous tumors (SFT) are mesenchymal neoplasms of soft tissue (commonly observed in the pleura). A study [28] on 28 SFT cases found that INSM1 was reactive in 21% (6/28) of the cases, which was slightly superior to desmin (14.3%, 4/28) and p16 (17.9%, 5/28), suggesting differential diagnosis of sarcomas can be supplemented by the use of the INSM1 marker. Specifically, when differentiating extraskeletal myxoid chondrosarcoma (EMC) from other mesenchymal tumors [25], INSM1 shows 90% positive staining with EMC but was negative with 94% of the other mesenchymal tumor samples of various cell types. Another study [26] that focused on a variety of sarcomas found that some angiosarcomas (26%, 24/94, mostly diffusely staining positive), a few desmoplastic small round cell tumors (11%, 7/62, weak to strong staining), and rarely some synovial sarcomas (4%, 3/76, moderate to strong staining) were positive for INSM1, while the other sarcomas including Ewing's sarcoma (0/57), clear cell sarcoma (0/14), soft tissue leiomyosarcoma (0/59), uterine leiomyosarcoma (0/65), alveolar soft part sarcoma (0/29), epithelioid sarcoma (0/30), and undifferentiated pleomorphic sarcoma (0/100) were negative [26].

In this study, we found that INSM1 might help in the differential diagnosis between AITL and Hodgkin's lymphoma. AITL originates from TFH cells (germinal center follicular helper T-cells) [29]. The most significant histological feature that distinguishes AITL from other peripheral T-cell lymphomas is the abundance of high endothelial microveins and clear cells in the tumor tissue [28]. The diagnosis of AITL requires the expression of at least two TFH markers (CD10, BCL6, PD1, ICOS, CXCL13, and CXCR5). Sometimes, the diagnosis of AITL may be confused with Hodgkin's lymphoma [29, 30]. We found that the mRNA and protein levels of INSM1 were significantly different between AITL and Hodgkin's lymphoma. INSM1 may contribute to diagnosis between AITL and Hodgkin's lymphoma, but more clinical practice is needed.

AITL is characterized by high frequency of epigenetic modification-related mutations such as TET2, IDH2, and DNMT3A [30]. Epigenetic therapy has proven to be a desirable option in AITL. Clinical studies have shown that the overall response rate (ORR) of the histone deacetylase inhibitors romidepsin or belinostat alone in AITL is 30% and 45%, respectively, higher than that of other T-cell lymphomas. The ORR was 75%, and the complete response (CR) rate was 42%, also higher than other subtypes, and all patients who benefit from epigenetic therapy had TET2 mutations. The combination of romidepsin and 5-azacytidine increased the ORR and CR of AITL to 83% and 50%, respectively [28–31].

INSM1 has a positive rate of nearly 50% in our samples. It is worth pondering whether INSM1 can become a unique marker of a certain subtype of AITL. Regrettably, we did not step further due to incomplete genetic molecular data in our patients. Several preclinical studies of INSM1-targeted therapies have shown promising results; then perhaps, patients who do not benefit from epigenetic therapy will benefit from INSM1-targeted therapies in the future.

Moreover, INSM1 may also help in the differential diagnosis between WDLPS and DDLPS. Genetic studies have shown that MDM2/CDK4 is the oncogene frequently amplified [32, 33]. MDM2 and CDK4 amplification can be seen in more than 90% of liposarcoma patients, but the karyotype and gene profile of DDLPS are far more complex than that of WDLPS [34].

Overexpression of INSM1 inhibits the binding of cyclin D1 and CDK4, induces cell cycle arrest, and inhibits the growth of panc-1 pancreatic cancer cell line [6]. INSM1 is an important transcription factor regulating the development of the nervous system and the differentiation of neural stem cells. The mechanism of interaction between INSM1 and CDK4 may be involved in the development of liposarcoma, which needs to be further explored.

Limitations of this study are as follows: firstly, the sample size of our study is relatively small. The findings remain further investigation to be more confirmed before clinic practice. Secondly, in diagnosis, it seems that other markers perform superiorly than INSM1. The differential diagnostical value of INSM1 seemed limited in particular cases. Finally, the function of INSM1 in non-NETs was not clear, and this study lacks deeper insights on how INSM1 expression influences the mesenchymal tumors.

## 5. Conclusion

Mesenchymal neoplasms, especially lymphomas and sarcomas, often progress rapidly, respond poorly to treatment, and have a poor prognosis. INSM1 is a reliable diagnosis marker in neuroendocrine tumors that has attracted much attention in recent years. Our study confirmed that there were statistically significant differences in the positive rate of INSM1 between AITL/Hodgkin's lymphomas and DDLPS/WDLPS, which may assist in the differential diagnosis of these tumors. What is more significant, targeted signaling pathways related to INSM1 contribute to the exploration of new treatment strategies. However, whether INSM1 can become a therapeutic target in mesenchymal tumors lacks theoretical and experimental bases. Here, our study systematically explored the expression of INSM1 in different mesenchymal tumors, laying a foundation for the diagnosis and targeted therapy of INSM1 in mesenchymal tumors.

## Figures and Tables

**Table 1:** The abbreviations of hematologic neoplasms mentioned in this study.

Tumor	Abbr
Diffuse large B-cell lymphoma	DLBCL
Follicular lymphoma	FL
Mantle cell lymphoma	MCL
Marginal zone lymphoma	MZL
Burkitt's lymphoma	BL
Hodgkin's lymphoma	HL
Plasmablastic lymphoma	PBL
Lymphoblastic lymphoma	LBL
NK/T-cell lymphoma	NKT
Small lymphocytic lymphoma	SLL
Anaplastic large cell lymphoma	ALCL
Angioimmunoblastic T-cell lymphoma	AITL

**Table 2:** INSM1 expression of hematologic neoplasms mentioned in this study.

Neoplasms histologic type	Number of positive cases/total cases (%)
Plasmablastic lymphoma	2/4 (50%)
Angioimmunoblastic T-cell lymphoma	18/38 (47.4%)
Burkitt's lymphoma	7/21 (33.3%)
Follicular lymphoma	9/28 (32.1%)
Marginal zone lymphoma	15/51 (29.4%)
Diffuse large B-cell lymphoma	5/19 (28.3%)
Mantle cell lymphoma	8/46 (17.4%)
T/B lymphoblastic lymphoma	4/33 (12.1%)
Hodgkin's lymphoma	1/14 (7.1%)
Small lymphocytic lymphoma	0/17 (0%)
NK/T-cell lymphoma	0/7 (0%)
Anaplastic large cell lymphoma	0/11 (0%)

**Table 3:** INSM1 expression of soft tissue neoplasms mentioned in this study.

Neoplasms histologic type	Number of positive cases/total cases (%)
Dedifferentiated liposarcoma	9/20 (45%)
Well-differentiated liposarcoma	2/21 (9.5%)
Myxoid liposarcoma	2/11 (18.2%)
Chondrosarcoma	3/6 (50%)
Ewing's sarcoma	4/13 (30.8%)
Osteogenic sarcoma	1/14 (7.1%)
Solitary fibrous tumor	4/9 (44.4%)
Dermatofibrosarcoma protuberans	0/22 (0%)
Myxoid fibrosarcoma	0/3 (0%)
Fibromyxoid sarcoma	0/3 (0%)
Rhabdomyosarcoma	13/49 (26.5%)
Granulosa cell tumor	1/19 (5.3%)
Undifferentiated sarcoma	2/40 (5%)
Gastrointestinal stromal tumor	0/11 (0%)
Hemangiosarcoma	0/14 (0%)
Leiomyosarcoma	0/7 (0%)
Synovial sarcoma	0/10 (0%)
Epithelioid sarcoma	0/8 (0%)
Alveolar soft part sarcoma	0/4 (0%)
Soft tissue clear cell sarcoma	0/3 (0%)

## Data Availability

The figure and table data used to support the findings of this study are included within the article and the supplementary information files.

## Supplementary Materials

Supplementary Materials Table S1: the selected dataset from GEO database. Table S2: lymphoma pairwise comparison. Table S3: soft tumor pairwise comparison. Figure S1: GEO analysis of INSM1 expression in bone tumors. normal_bonechon: normal bone tissue; oste: osteosarcoma; chon: chondrosarcoma; EWS: Ewing's sarcoma. Figure S2: GEO analysis of INSM1 expression in rhabdomyosarcoma. normal_SKMU: normal skeletal muscle; rha: rhabdomyosarcoma. Figure S3: expression of INSM1 in MZL/DLBCL/MCL/LBL. (A, B) Marginal zone lymphoma (MZL): cell composition is diverse with lymphocytes, as well as central and immunoblasts, INSM1+ (200x); (C, D) diffuse large B-cell lymphoma (DLBCL): large to moderate tumor cells with ovoid nuclei and blastocytic INSM1+ (400x); (E, F) mantle cell lymphoma (MCL): neoplasms are moderately round in size, densely packed, with little cytoplasm, single-scattered epithelioid cells, and hyaline vascular INSM1+ (400x); (G, H) lymphoblastic lymphoma: diffuse distribution of tumor cells, single component, similar morphology, high nucleo-plasma ratio INSM1 (400x). Figure S4: expression of INSM1 in rhabdomyosarcoma/undifferentiated sarcoma/leiomyosarcoma/angiosarcoma. Rhabdomyosarcoma (A, B): the tumor cells are patchy in distribution, with dark nuclei, round or oval nuclei, mitotic images, sparse cytoplasm, and light staining. INSM1+ (400x); (C, D) undifferentiated sarcomas: numerous pleomorphic cells, pathologic mitotic INSM1+ (400x); (E, F) leiomyosarcoma: spindular cells arranged in a woven pattern, abundant cytoplasm, INSM1- (200x) in the center of the nucleus; (G, H) hemangiosarcoma: large round cells arranged in sheets or cords, with abundant sinuses in the tumor area (400x). Figure S5: expression of INSM1 in fibroblastic/myofibroblastic tumors. Solitary fibrous tumors (A, B): the cells were oval or spindle shaped. The cytoplasm in the central area was mainly eosinophilic, and the cytoplasm in the peripheral area was bright with collagen interspersed, INSM1+ (400x). (C, D) Dermatofibrosarcoma protuberans: the tumor area consists of spindle-shaped cells arranged in a mat pattern, INSM1- (400x); (E, F) Myxofibrosarcoma: the tumor cells are abundant in bundles, the nuclei are varied in shape, and giant cells can be seen, INSM1- (400x); (G, H) fibromyxoid sarcoma: microscopically, numerous fibrous structures arranged in interlocking bundles are interspersed with round/spindle nuclei. Mitograms are rare. INSM1- (400x). Figure S6: INSM1 expression in some undetermined differentiated tumors. Synovial sarcomas (A, B): the neoplasm is of high cell density, with collagen fibers between spindle cells and cells arranged in bundles. The cytoplasm of the epithelioid cells was rich, light, and bright, with clear intercellular boundaries (400x). (C, D) Epithelioid sarcomas: eosinophilic epithelioid and spindle cells with vacuolar nuclei. Collagen deposition was observed between cells (400x). (E, F) Alveolar soft tissue sarcoma: the tumor cells were arranged “organ-like” with fibrous interspersed with different widths (400x); (G, H) clear cell sarcoma of soft tissue: the tumor cells are polygonal and oval, with faint cytoplasm and scattered hyaline areas, with large myxoid areas at the right. INSM1 staining in the figure was negative (400x). Figure S7: positive expression of INSM1 in bone tumors. Chondrosarcoma (A, D): tumor cells were scattered in the myxoid matrix, and the nuclei of tumor cells were large and dark with different shapes. Osteosarcomas (B, E) are braided neoplastic cells, mainly spindle shaped, with pleomorphic cells also seen. Osteoid matrix is not evident. (C, F) Ewing's sarcoma: microscopically, Ewing's sarcoma consists of homogenous small round cells with fine chromatin and sparse transparent or faintly stained cytoplasm (400x).
