# Supplementary material for: INSM1 Expression in Mesenchymal Tumors and Its Clinicopathological Significance
Source: Biomed Res Int. 2022 Dec 7;2022:1580410. doi: 10.1155/2022/1580410 (PMC9750778; doi:10.1155/2022/1580410)
Supplement: Supplementary Materials — Table S1: the selected dataset from GEO database. Table S2: lymphoma pairwise comparison. Table S3: soft tumor pairwise comparison. Figure S1: GEO analysis of INSM1 expression in bone tumors. normal_bonechon: normal bone tissue; oste: osteosarcoma; chon: chondrosarcoma; EWS: Ewing's sarcoma. Figure S2: GEO analysis of INSM1 expression in rhabdomyosarcoma. normal_SKMU: normal skeletal muscle; rha: rhabdomyosarcoma. Figure S3: expression of INSM1 in MZL/DLBCL/MCL/LBL. (A, B) Marginal zone lymphoma (MZL): cell composition is diverse with lymphocytes, as well as central and immunoblasts, INSM1+ (200x); (C, D) diffuse large B-cell lymphoma (DLBCL): large to moderate tumor cells with ovoid nuclei and blastocytic INSM1+ (400x); (E, F) mantle cell lymphoma (MCL): neoplasms are moderately round in size, densely packed, with little cytoplasm, single-scattered epithelioid cells, and hyaline vascular INSM1+ (400x); (G, H) lymphoblastic lymphoma: diffuse distribution of tumor cells, single component, similar morphology, high nucleo-plasma ratio INSM1 (400x). Figure S4: expression of INSM1 in rhabdomyosarcoma/undifferentiated sarcoma/leiomyosarcoma/angiosarcoma. Rhabdomyosarcoma (A, B): the tumor cells are patchy in distribution, with dark nuclei, round or oval nuclei, mitotic images, sparse cytoplasm, and light staining. INSM1+ (400x); (C, D) undifferentiated sarcomas: numerous pleomorphic cells, pathologic mitotic INSM1+ (400x); (E, F) leiomyosarcoma: spindular cells arranged in a woven pattern, abundant cytoplasm, INSM1- (200x) in the center of the nucleus; (G, H) hemangiosarcoma: large round cells arranged in sheets or cords, with abundant sinuses in the tumor area (400x). Figure S5: expression of INSM1 in fibroblastic/myofibroblastic tumors. Solitary fibrous tumors (A, B): the cells were oval or spindle shaped. The cytoplasm in the central area was mainly eosinophilic, and the cytoplasm in the peripheral area was bright with collagen interspersed, INSM1+ (400x). (C, D) Derma [file 1580410.f1.docx]

# INSM1 expression in mesenchymal tumors and its clinicopathological significance

## Supplementary Materials

**Table S1**

The selected data set from GEO database

|  | **title** | **label** |  | **title** | **label** |
| --- | --- | --- | --- | --- | --- |
| 1 | BL | GSE26673 | 44 | PLS | GSE20559 |
| 2 | BL | GSE68950 | 45 | PLS | GSE21122 |
| 3 | BL | GSE29492 | 46 | PLS | GSE21124 |
| 4 | SLL | GSE22762 | 47 | oste | GSE11127 |
| 5 | SLL | GSE9327 | 48 | oste | GSE14827 |
| 6 | DLBCL | GSE10846 | 49 | oste | GSE16091 |
| 7 | FL | GSE16455 | 50 | ASPS | GSE13433 |
| 8 | FL | GSE9327 | 51 | ASPS | GSE49327 |
| 9 | MZL | GSE32018 | 52 | EWS | GSE17618 |
| 10 | MZL | GSE32231 | 53 | EWS | GSE37371 |
| 11 | MZL | GSE9327 | 54 | SS | GSE20196 |
| 12 | MCL | GSE36000 | 55 | SS | GSE2719 |
| 13 | MCL | GSE70910 | 56 | SS | GSE35852 |
| 14 | MCL | GSE70927 | 57 | SS | GSE54187 |
| 15 | MCL | GSE9327 | 58 | LMS | GSE21050 |
| 16 | AITL | GSE19069 | 59 | MFS | GSE21122 |
| 17 | ALCL | GSE19069 | 60 | UDS | GSE21050 |
| 18 | LBL | GSE10820 | 61 | AGS | GSE44115 |
| 19 | LBL | GSE28462 | 62 | GIST | GSE8167 |
| 20 | LBL | GSE62006 | 63 | GIST | GSE17743 |
| 21 | NKT | GSE20874 | 64 | GIST | GSE13861 |
| 22 | PBL | GSE53117 | 65 | GIST | GSE2719 |
| 23 | HL | GSE7788 | 66 | rha | GSE66533 |
| 24 | HL | GSE66728 | 67 | rha | GSE68015 |
| 25 | HL | GSE25990 | 68 | rha | GSE967 |
| 26 | NKT | GSE58445 | 69 | rha | GSE28511 |
| 27 | MLS | GSE20559 | 70 | chon | GSE12532 |
| 28 | MLS | GSE30929 | 71 | chon | GSE30835 |
| 29 | MLS | GSE62747 | 72 | chon | GSE30844 |
| 30 | MLS | GSE55466 | 73 | SF | GSE43045 |
| 31 | MLS | GSE55466 | 74 | CCS | GSE43046 |
| 32 | MLS | GSE6481 | 75 | CCS | GSE43047 |
| 33 | DDLPS | GSE20559 | 76 | CCS | GSE43048 |
| 34 | DDLPS | GSE21050 | 77 | CCS | GSE2553 |
| 35 | WDLPS | GSE20559 | 78 | GCT | GSE72035 |
| 36 | normal_blood | GSE72028 | 79 | normal_bonechon | GSE28425 |
| 37 | normal_blood | GSE55851 | 80 | normal_bonechon | GSE36001 |
| 38 | normal_blood | GSE65136 | 81 | normal_bonechon | GSE9508 |
| 39 | normal_LN | GSE32018 | 82 | normal_bonechon | GSE30835 |
| 40 | normal_LN | GSE32231 | 83 | normal_SKMU | GSE17674 |
| 41 | normal_LN | GSE29492 | 84 | normal_SKMU | GSE2719 |
| 42 | normal_fat | GSE21122 | 85 | normal_SKMU | GSE44115 |
| 43 | normal_fat | GSE62747 |  |  |  |

**Table S2**

Lymphoma pairwise comparison

|  | **V1** | **V2** | **ttest.p** |  | | **V1** | **V2** | **ttest.p** |
| --- | --- | --- | --- | --- | --- | --- | --- | --- |
| 1 | PBL | NKT | 0.679922 | | 34 | BL | FL | 0.333951 |
| 2 | PBL | LBL | 0.815364 | | 35 | BL | MZL | 0.540839 |
| 3 | PBL | BL | 0.979695 | | 36 | BL | DLBCL | 0.668614 |
| 4 | PBL | MCL | 0.452623 | | 37 | BL | ALCL | 0.045151* |
| 5 | PBL | HL | 0.639781 | | 38 | BL | AITL | 2.27E-05* |
| 6 | PBL | SLL | 0.832615 | | 39 | MCL | HL | 0.729661 |
| 7 | PBL | FL | 0.576171 | | 40 | MCL | SLL | 0.438274 |
| 8 | PBL | MZL | 0.723344 | | 41 | MCL | FL | 0.69062 |
| 9 | PBL | DLBCL | 0.813679 | | 42 | MCL | MZL | 0.490505 |
| 10 | PBL | ALCL | 0.134068 | | 43 | MCL | DLBCL | 0.179618 |
| 11 | PBL | AITL | 0.012324* | | 44 | MCL | ALCL | 0.33843 |
| 12 | NKT | LBL | 0.748708 | | 45 | MCL | AITL | 1.91E-05* |
| 13 | NKT | BL | 0.603229 | | 46 | HL | SLL | 0.70443 |
| 14 | NKT | MCL | 0.268743 | | 47 | HL | FL | 0.974178 |
| 15 | NKT | HL | 0.38762 | | 48 | HL | MZL | 0.803093 |
| 16 | NKT | SLL | 0.504416 | | 49 | HL | DLBCL | 0.345896 |
| 17 | NKT | FL | 0.333694 | | 50 | HL | ALCL | 0.206145 |
| 18 | NKT | MZL | 0.422557 | | 51 | HL | AITL | 0.000275* |
| 19 | NKT | DLBCL | 0.773734 | | 52 | SLL | FL | 0.575299 |
| 20 | NKT | ALCL | 0.082759 | | 53 | SLL | MZL | 0.826395 |
| 21 | NKT | AITL | 0.084385 | | 54 | SLL | DLBCL | 0.493408 |
| 22 | LBL | BL | 0.612881 | | 55 | SLL | ALCL | 0.082281 |
| 23 | LBL | MCL | 0.134333 | | 56 | SLL | AITL | 1.67E-05* |
| 24 | LBL | HL | 0.299614 | | 57 | FL | MZL | 0.663304 |
| 25 | LBL | SLL | 0.421417 | | 58 | FL | DLBCL | 0.181052 |
| 26 | LBL | FL | 0.083345 | | 59 | FL | ALCL | 0.140755 |
| 27 | LBL | MZL | 0.174505 | | 60 | FL | AITL | 1.49E-07* |
| 28 | LBL | DLBCL | 0.971325 | | 61 | MZL | DLBCL | 0.305213 |
| 29 | LBL | ALCL | 0.016576* | | 62 | MZL | ALCL | 0.083558 |
| 30 | LBL | AITL | 7.20E-06* | | 63 | MZL | AITL | 2.81E-07* |
| 31 | BL | MCL | 0.28963 | | 64 | DLBCL | ALCL | 0.026332* |
| 32 | BL | HL | 0.51997 | | 65 | DLBCL | AITL | 0.00021* |
| 33 | BL | SLL | 0.759738 | | 66 | ALCL | AITL | 4.87E-06* |

*P<0.05

**Table S3**

Soft tumors pairwise comparison

|  | **V1** | **V2** | **ttest.p** |  | **V1** | **V2** | **ttest.p** |
| --- | --- | --- | --- | --- | --- | --- | --- |
| 1 | SF | AGS | 0.416415 | 69 | ASPS | rha | 0.621957 |
| 2 | SF | ASPS | 0.782201 | 70 | ASPS | GCT | 0.339034 |
| 3 | SF | SS | 0.600364 | 71 | ASPS | GIST | 0.714134 |
| 4 | SF | MLS | 0.388119 | 72 | ASPS | chon | 0.000183* |
| 5 | SF | rha | 0.954713 | 73 | ASPS | oste | 0.00172* |
| 6 | SF | GCT | 0.833354 | 74 | ASPS | EWS | 0.019355* |
| 7 | SF | GIST | 0.896266 | 75 | ASPS | DDLPS | 0.116705 |
| 8 | SF | chon | 0.133389 | 76 | ASPS | WDLPS | 0.110218 |
| 9 | SF | oste | 0.183367 | 77 | ASPS | PLS | 0.120211 |
| 10 | SF | EWS | 0.183998 | 78 | ASPS | LMS | 0.609561 |
| 11 | SF | DDLPS | 0.367277 | 79 | ASPS | UDS | 0.000546* |
| 12 | SF | WDLPS | 0.360419 | 80 | ASPS | MFS | 0.003951* |
| 13 | SF | PLS | 0.338766 | 81 | SS | MLS | 0.001833* |
| 14 | SF | LMS | 0.959485 | 82 | SS | rha | 0.516708 |
| 15 | SF | UDS | 0.162443 | 83 | SS | GCT | 0.571812 |
| 16 | SF | MFS | 0.168856 | 84 | SS | GIST | 0.165232 |
| 17 | AGS | ASPS | 0.290775 | 85 | SS | chon | 2.08E-06* |
| 18 | AGS | SS | 0.035587 | 86 | SS | oste | 2.13E-05* |
| 19 | AGS | MLS | 0.986368 | 87 | SS | EWS | 0.000536* |
| 20 | AGS | rha | 0.222798 | 88 | SS | DDLPS | 0.003225* |
| 21 | AGS | GCT | 0.096853 | 89 | SS | WDLPS | 0.506719 |
| 22 | AGS | GIST | 0.192888 | 90 | SS | PLS | 0.004585* |
| 23 | AGS | chon | 0.194592 | 91 | SS | LMS | 0.488157 |
| 24 | AGS | oste | 0.302394 | 92 | SS | UDS | 6.25E-06* |
| 25 | AGS | EWS | 0.3162 | 93 | SS | MFS | 6.06E-05* |
| 26 | AGS | DDLPS | 0.895895 | 94 | MLS | rha | 0.154558 |
| 27 | AGS | WDLPS | 0.037824* | 95 | MLS | GCT | 0.027531* |
| 28 | AGS | PLS | 0.791868 | 96 | MLS | GIST | 0.023937* |
| 29 | AGS | LMS | 0.206335 | 97 | MLS | chon | 0.000197* |
| 30 | AGS | UDS | 0.25811 | 98 | MLS | oste | 0.018655* |
| 31 | AGS | MFS | 0.261003 | 99 | MLS | EWS | 0.174102 |
| 32 | ASPS | SS | 0.104386 | 100 | MLS | DDLPS | 0.858317 |
| 33 | ASPS | MLS | 0.09595 | 101 | MLS | WDLPS | 0.024802* |
| 34 | MLS | PLS | 0.727814 | 102 | chon | EWS | 0.698495 |
| 35 | MLS | LMS | 0.131301 | 103 | chon | DDLPS | 0.020619* |
| 36 | MLS | UDS | 0.001344* | 104 | chon | WDLPS | 0.003633* |
| 37 | MLS | MFS | 0.056812 | 105 | chon | PLS | 0.10196 |
| 38 | rha | GCT | 0.836664 | 106 | chon | LMS | 0.006408 |
| 39 | rha | GIST | 0.775451 | 107 | chon | UDS | 0.104304 |
| 40 | rha | chon | 0.010487* | 108 | chon | MFS | 0.620798 |
| 41 | rha | oste | 0.026106* | 109 | oste | EWS | 0.892504 |
| 42 | rha | EWS | 0.042024* | 110 | oste | DDLPS | 0.131554 |
| 43 | rha | DDLPS | 0.150297 | 111 | oste | WDLPS | 0.006372* |
| 44 | rha | WDLPS | 0.290886 | 112 | oste | PLS | 0.303304 |
| 45 | rha | PLS | 0.13853 | 113 | oste | LMS | 0.017988* |
| 46 | rha | LMS | 0.99256 | 114 | oste | UDS | 0.597567 |
| 47 | rha | UDS | 0.01762* | 115 | oste | MFS | 0.795006 |
| 48 | rha | MFS | 0.026134* | 116 | EWS | DDLPS | 0.272948 |
| 49 | GCT | GIST | 0.485032 | 117 | EWS | WDLPS | 0.007647* |
| 50 | GCT | chon | 0.000987* | 118 | EWS | PLS | 0.394529 |
| 51 | GCT | oste | 0.0024* | 119 | EWS | LMS | 0.03326* |
| 52 | GCT | EWS | 0.005712* | 120 | EWS | UDS | 0.959702 |
| 53 | GCT | DDLPS | 0.030251* | 121 | EWS | MFS | 0.965292 |
| 54 | GCT | WDLPS | 0.31097 | 122 | DDLPS | WDLPS | 0.023904* |
| 55 | GCT | PLS | 0.031271* | 123 | DDLPS | PLS | 0.861154 |
| 56 | GCT | LMS | 0.81982 | 124 | DDLPS | LMS | 0.12968 |
| 57 | GCT | UDS | 0.00173* | 125 | DDLPS | UDS | 0.056851 |
| 58 | GCT | MFS | 0.002323* | 126 | DDLPS | MFS | 0.152835 |
| 59 | GIST | chon | 4.46E-06* | 127 | WDLPS | PLS | 0.022087* |
| 60 | GIST | oste | 0.000103* | 128 | WDLPS | LMS | 0.275547 |
| 61 | GIST | EWS | 0.006375* | 129 | WDLPS | UDS | 0.005089* |
| 62 | GIST | DDLPS | 0.041322* | 130 | WDLPS | MFS | 0.005834* |
| 63 | GIST | WDLPS | 0.145529 | 131 | PLS | LMS | 0.120565 |
| 64 | GIST | PLS | 0.051403* | 132 | PLS | UDS | 0.195299 |
| 65 | GIST | LMS | 0.771181 | 133 | PLS | MFS | 0.286771 |
| 66 | GIST | UDS | 1.78E-05* | 134 | LMS | UDS | 0.011417* |
| 67 | GIST | MFS | 0.000601* | 135 | LMS | MFS | 0.018751* |
| 68 | chon | oste | 0.17379 | 136 | UDS | MFS | 0.998459 |

*P< 0.05


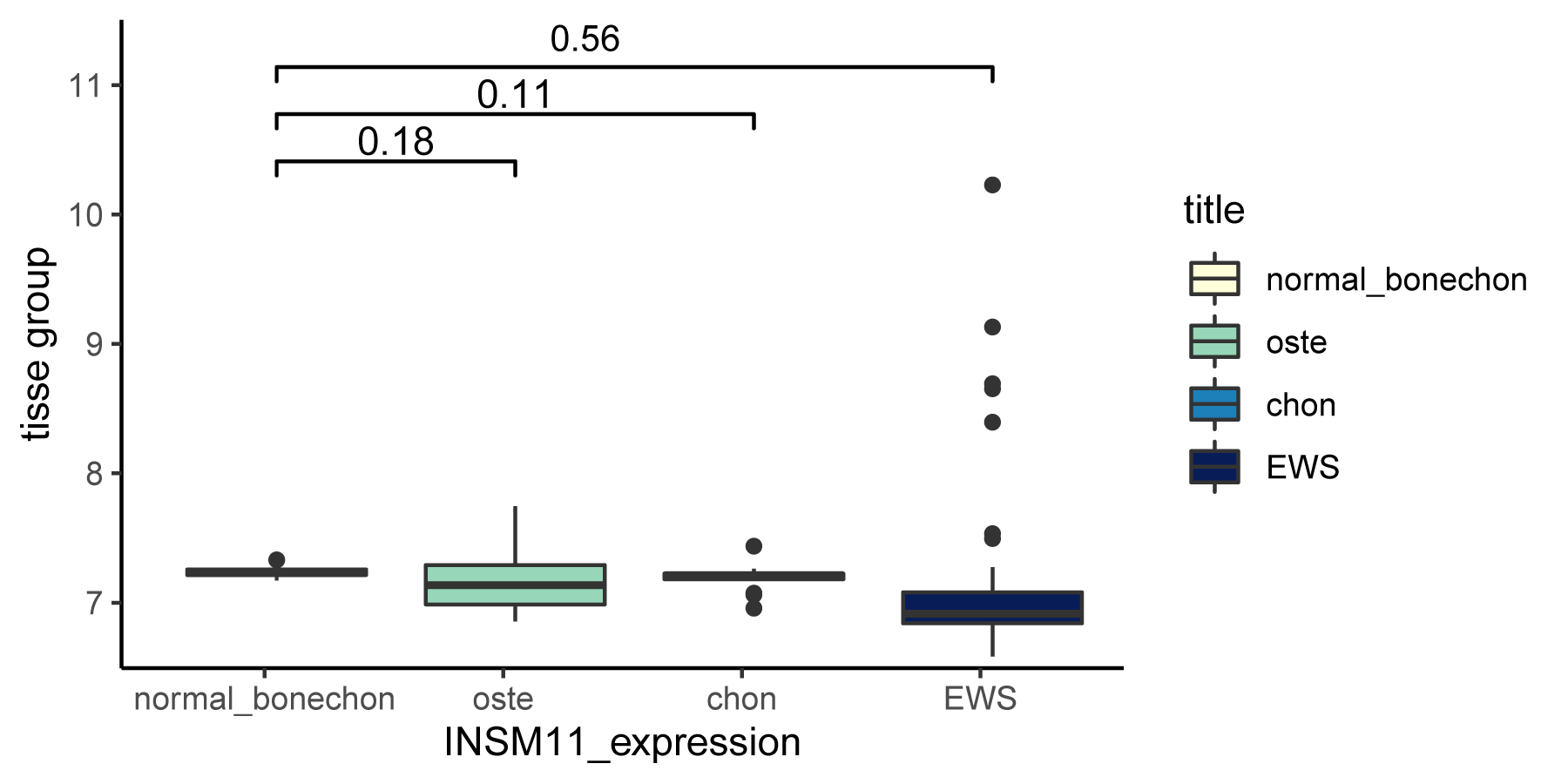


**Figure S1**

GEO analysis of INSM1 expression in bone tumors

normal_bonechon: Normal bone tissue, oste: osteosarcoma, chon: chondrosarcoma; EWS: ewing sarcoma


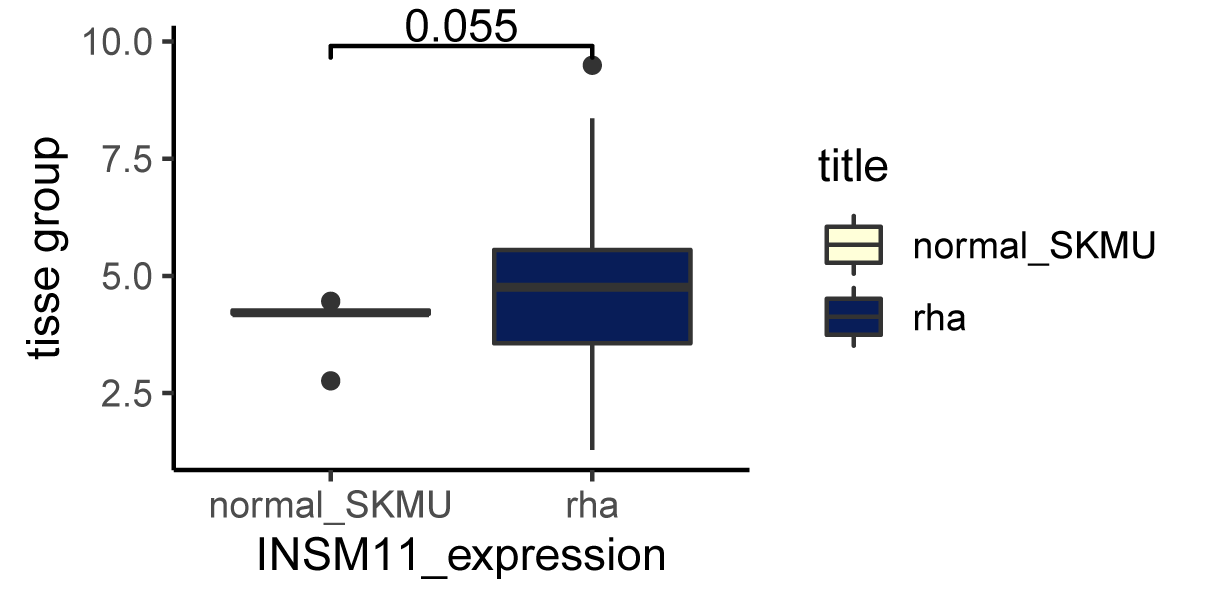


**Figure S2**

GEO analysis of INSM1 expression in rhabdomyosarcoma

normal_SKMU: Normal skeletal muscle, rha: rhabdomyosarcoma


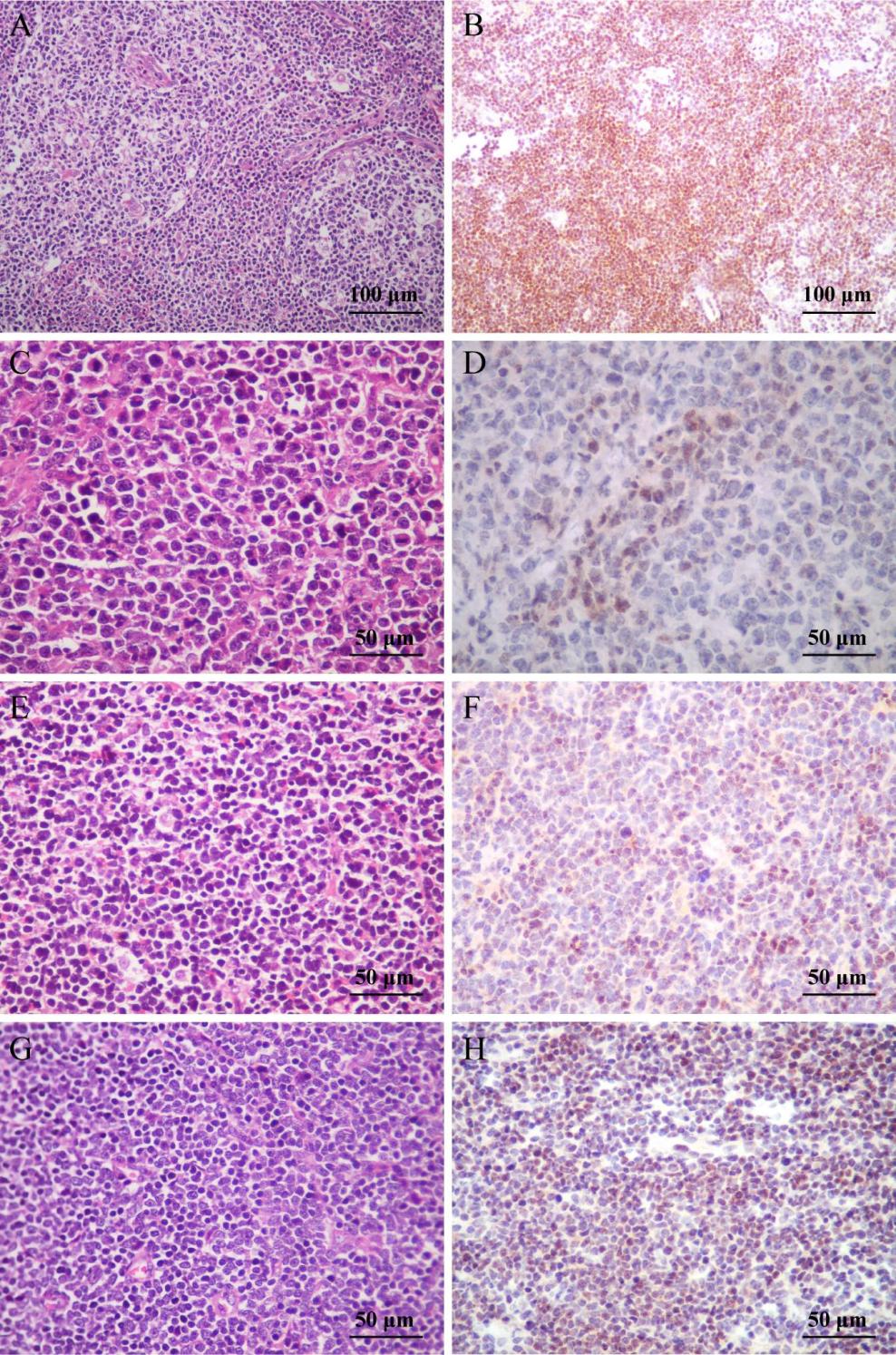


**Figure S3** Expression of INSM1 in MZL /DLBCL/MCL/LBL

A and B Marginal zone lymphoma (MZL) : Cell composition is diverse with lymphocytes, as well as central and immunoblasts, INSM1+ (200×); C and D Diffuse Large B-cell lymphoma (DLBCL) : large to moderate tumor cells with ovoid nuclei an d blastocytic INSM1+ (400×); E and F mantle cell lymphoma (MCL) : Neoplasms are moderately round in size, densely packed, with little cytoplasm, single scattered epithelioid cells, and hyaline vascular INSM1+ (400×). G and H: Lymphoblastic lymphoma: diffuse distribution of tumor cells, single component, similar morphology, high nucleo-plasma ratio INSM1 (400×);

**
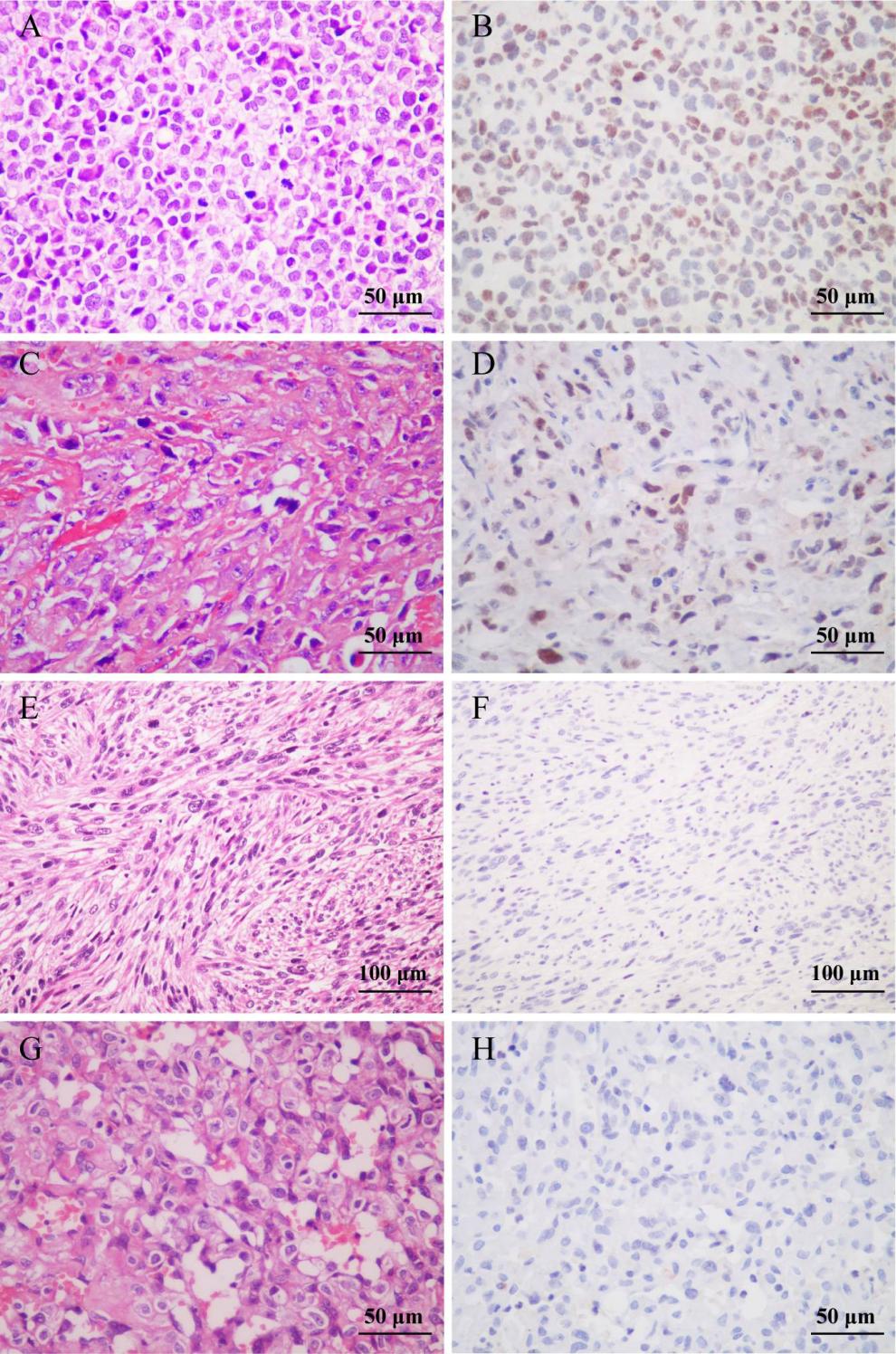
**

**Figure S4** Expression of INSM1 in rhabdomyosarcoma/undifferentiated sarcoma/leiomyosarcoma/angiosarcoma

Rhabdomyosarcoma A and B: The tumor cells are patchy in distribution, with dark nuclei, round or oval nuclei, mitotic images, sparse cytoplasm and light staining. INSM1+ (400×); C, D undifferentiated sarcomas: numerous pleomorphic cells, pathologic mitotic INSM1+ (400×); E, F: Leiomyosarcoma: spindular cells arranged in a woven pattern, abundant cytoplasm, INSM1- (200×) in the center of the nucleus; G, H Hemangiosarcoma: large round cells arranged in sheets or cords, with abundant sinuses in the tumor area (400 x)

**
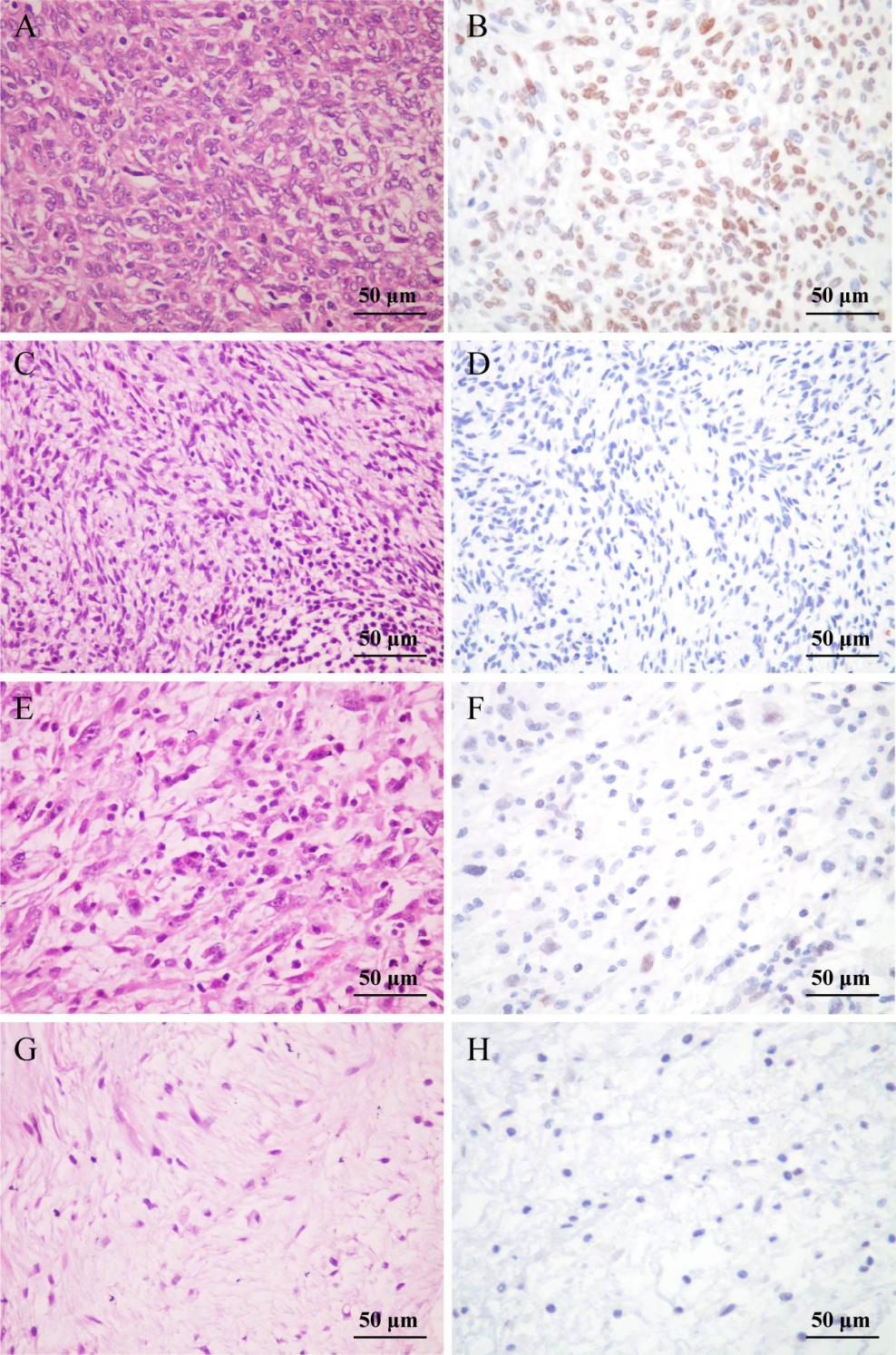
**

**Figure S5** Expression of INSM1 in fibroblastic/myofibroblastic tumors

Isolated fibrous tumors A and B: the cells were oval or spindle shaped. The cytoplasm in the central area was mainly eosinophilic, and the cytoplasm in the peripheral area was bright with collagen interspersed, INSM1+ (400×). C, D Dermatofibrosarcoma protuberans: The tumor area consists of spindle-shaped cells arranged in a mat pattern, INSM1- (400×); E, F myxomyosarcoma: the tumor cells are abundant in bundles, the nuclei are varied in shape, giant cells can be seen, INSM1- (400×); G, H fibromyxoid sarcoma: Microscopically, numerous fibrous structures arranged in interlocking bundles are interspersed with round/spindle nuclei. Mitograms are rare. INSM1 -; (400 x)

**
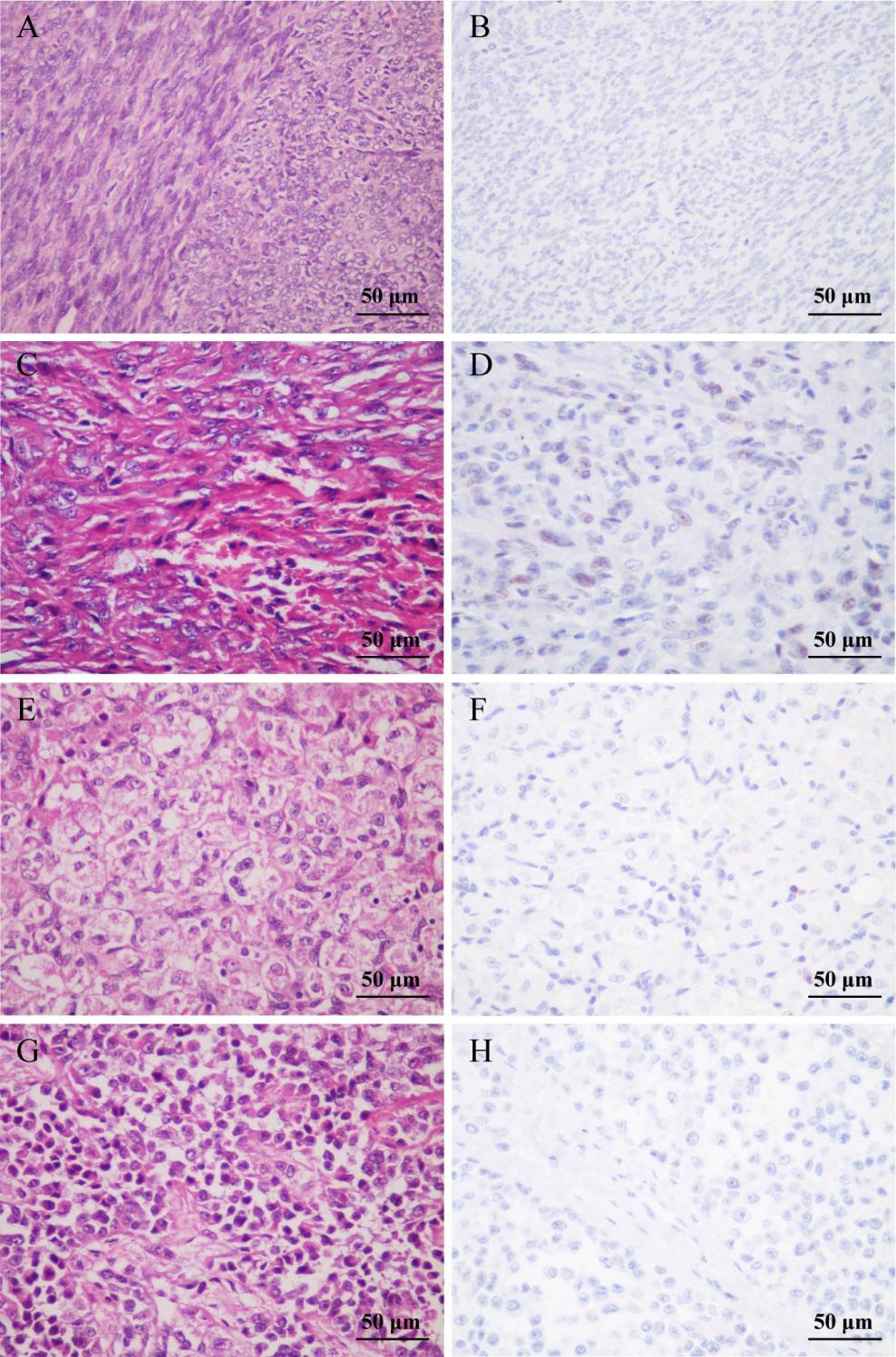
**

**Figure S6** INSM1 expression in some undetermined differentiated tumors

Synovial sarcomas A and B: The neoplasm is of high cell density, with collagen fibers between spindle cells and cells arranged in bundles. The cytoplasm of the epithelioid cells was rich, light and bright, with clear intercellular boundaries (400×). C, D Epithelioid sarcomas: eosinophilic epithelioid and spindle cells with vacuolar nuclei. Collagen deposition was observed between cells (400×). E, F alveolar soft tissue sarcoma: the tumor cells were arranged "organ-like" with fibrous interspersed with different widths (400×); G, H Clear cell sarcoma of soft tissue: The tumor cells are polygonal and oval, with faint cytoplasm and scattered hyaline areas, with large myxoid areas at the right. INSM1 staining in the figure was negative (400×)


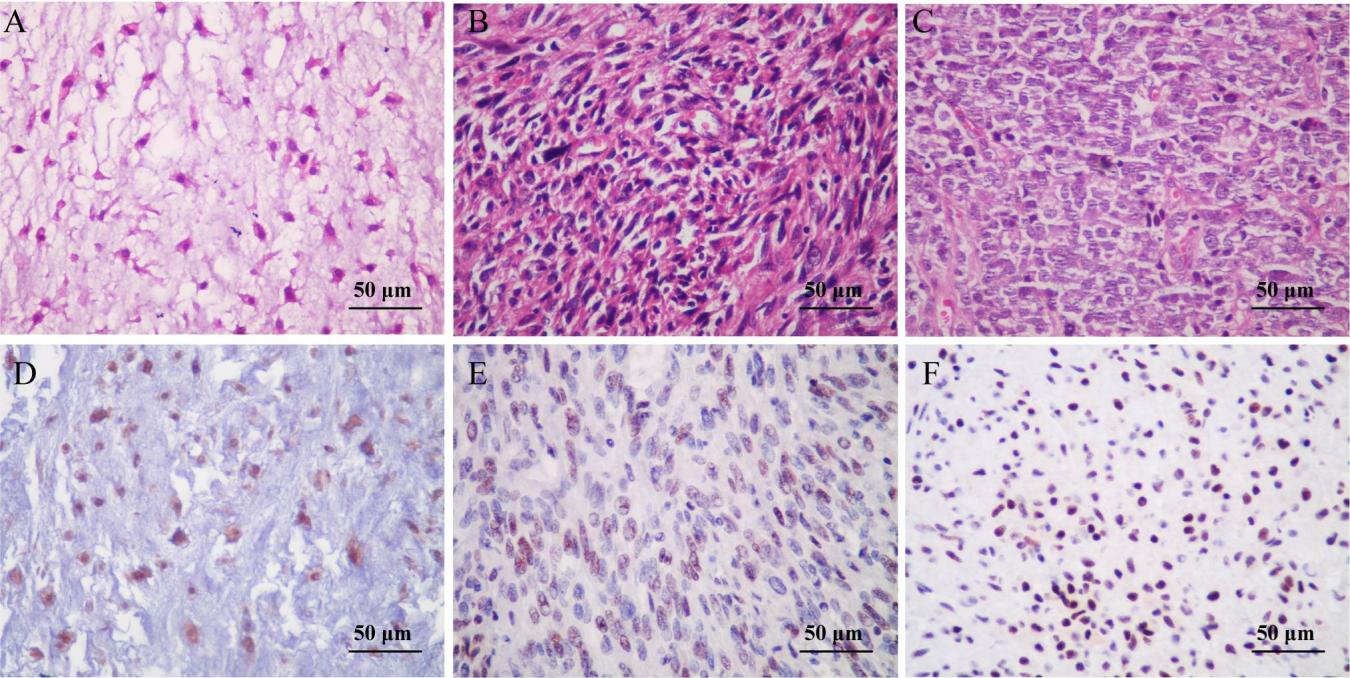


**Figure S7** Positive expression of INSM1 in bone tumors

Chondrosarcoma A and D: tumor cells were scattered in the myxoid matrix, and the nuclei of tumor cells were large and dark with different shapes. Osteosarcomas B and E are braided neoplastic cells, mainly spindle shaped, with pleomorphic cells also seen. Osteoid matrix is not evident. C, F Ewing's sarcoma: microscopically, Ewing's sarcoma consists of homogenous small round cells with fine chromatin and sparse transparent or faintly stained cytoplasm. (400 x)
